# Predictive value of the aspartate aminotransferase to platelet ratio index for parenteral nutrition associated cholestasis in extremely low birth weight infants

**DOI:** 10.1186/s12887-019-1493-8

**Published:** 2019-04-24

**Authors:** Ji Hye Hwang, Mi Lim Chung

**Affiliations:** Department of Pediatrics, Haeundae Paik Hospital, Inje University College of Medicine, 875, Haeundaero, Haeundae-gu, Busan, 48108 Korea

**Keywords:** Aspartate aminotransferase to platelet ratio index, Parenteral nutrition, Parenteral nutrition-associated cholestasis, Extremely low birth weight infants

## Abstract

**Background:**

Parenteral nutrition (PN) improves the survival of premature infants. However, prolonged PN increases the risk of PN-associated cholestasis (PNAC).

**Objective:**

We aimed to evaluate the predictive value of aspartate aminotransferase (AST)-to-platelet ratio index (APRI) for PNAC in infants with extremely low birth weight (ELBW, birth weight < 1000 g) infants.

**Methods:**

We retrospectively reviewed the medical records of ELBW infants from March 2010 to February 2017. Clinical data and the serial APRI, AST, alanine aminotransferase (ALT), AST-to-ALT ratio, and direct bilirubin (DB) were analyzed. PNAC was diagnosed in infants with a history of PN for at least 2 weeks and direct bilirubin concentrations > 2 mg/dL after other causes of neonatal cholestasis were excluded.

**Results:**

Among the 179 eligible ELBW infants, 56 (31.3%) were diagnosed with PNAC. APRI significantly differed between infants with PNAC and those without PNAC. The best APRI cut-off point was 0.410 at 2 weeks after the start of PN (area under the receiver operating characteristic curve = 0.752, *p < 0.05*; positive predictive value, 50.6%; negative predictive value, 84.1%).

**Conclusion:**

APRI at 2 weeks after PN could be a reliable predictor of PNAC development in ELBW infants on PN.

## Background

Parenteral nutrition (PN) is essential for improving the growth and development of premature infants before enteral feeding can be established. However, long-term PN can also increase the risk of various PN-associated hepatobiliary complications [1, 2]. PN-associated cholestasis (PNAC) is the most common clinical manifestation of PN-associated liver disease in preterm infants. The pathogenesis of PNAC is considered multifactorial. Several identified risk factors associated with PNAC are known; prematurity, small for gestational age, long duration of PN, sepsis, necrotizing enterocolitis (NEC), composition of PN solutions and a delay in enteral feeding [3, 4]. Though most cases of PNAC resolve with enteral nutrition [5], progressive hepatic failure eventually can lead to death in some patients. Therefore, early identification of groups at risk of PNAC helps with an earlier adjustment in PN to decrease the risk of progressive to severe liver dysfunction.

The diagnosis of PNAC is made by checking the increased levels of direct bilirubin (DB). It is very simple and inexpensive. However, there is a limit to predict the development of PNAC by tracking values of DB alone. In many patients, the level of DB did not increase gradually. Even, it suddenly increase up to the point to diagnose PNAC. Therefore, we sought to find a new way to predict the PNAC. Numerous studies have demonstrated that the aspartate aminotransferase (AST)-to-platelet ratio index (APRI) is a reliable marker of liver fibrosis in adult patients [6, 7]. Although studies are limited, the APRI has been investigated as a marker of hepatic fibrosis in chronic liver disease, hepatitis, and biliary atresia in the younger population [8–11]. The histopathology of PNAC has been described as a progression from bile duct proliferation to portal inflammation to bridging fibrosis to cirrhosis [12, 13]. Hence, we assumed that APRI also can be applied in PNAC of neonates. Only one study evaluated the predictive value of APRI for PNAC in premature infants, to date [14] and it enrolled a limited number of preterm infants with intestinal perforation.

This study was conducted to evaluate the predictive value of the APRI for PNAC in infants with extremely low birth weight (ELBW).

## Methods

### Subjects

We reviewed the medical records of ELBW infants, with a birth weight of less than 1000 g, who were admitted to the NICU at Haeundae Paik Hospital, Busan, Korea between March 2010 and February 2018. The inclusion criteria were ELBW infants who received PN for at least 2 weeks and survived for more than 4 weeks. We excluded infants who had severe congenital anomalies, chromosomal abnormalities or clinically apparent congenital infections. Infants with other causes of cholestasis including hypothyroidism, gallstone were excluded and infants with congenital platelet disorder were excluded. We also excluded infants who were transferred to other hospital.

### Methods

PNAC was defined as those with a direct bilirubin concentration > 2.0 mg/dL without other identifiable causes of cholestasis except for PN. We excluded the data when there was a temporary increase in DB accompanying sepsis and shortly resolved at follow up tests.

We collected basic demographic data and reviewed clinical variables including respiratory distress syndrome, patent ductus arteriosus, NEC, sepsis, transfusion history, surgery, ventilator care duration, and brochopulmonary dysplasia (BPD). NEC was defined as Bell’s criteria stage two or greater. BPD was defined as a need for oxygen at 36 weeks of postmenstrual age. Maternal data were also reviewed. Data of nutritional support were also collected including the number of days until the initiation of enteral feeding, the time points at which half (60 cc/kg/day) and full (120 cc/kg/day) enteral feeding were reached and the number of days of total PN, and types of PN solutions.

The laboratory findings were reviewed to determine levels of total bilirubin (TB), DB, and liver enzymes including AST and alanine aminotransferase (ALT). The APRI was calculated from the equation according to Wai et al. [15] as follows: (AST/upper normal limit of AST)/platelet count (10^9^/L) *100, and upper normal limit of AST value was defined as 40 U/L. We analyzed laboratory results in each time point as 1, 2, and 3 weeks after starting PN.

### Nutrition protocol

Though it was varied according to the change of guidelines of our unit and the tolerance of the individual patient, common nutritional supplementation principle was as following and this methodology was used also from a previously work [16].

#### Enteral nutrition protocol

The starting enteral feeding volume was 10–20 mL/kg/day divided into doses administered every 3 h and increased by 10–20 mL/kg/day according to the baby’s tolerance until full enteral feeding (120–160 mL/kg/day) was achieved. We fed the ELBW infants either human milk or premature infant formula. After the enteral feeding volume reached 100 mL/kg/day, breastmilk was fortified with a human milk fortifier.

#### Parenteral nutrition protocol

##### Proteins

The infants were started at 0.5 g/kg just after birth. The dose was increased by 0.5 g/kg/day increments every day to a maximum dose of 3.0–4.0 g/kg/day according to the baby’s tolerance. From 2016, we administered 1.5 g/kg/day as starting dose of parenteral amino acid and increased rapidly by 1.0 g/kg/day to reach maximum concentration of 4.0–4.5 g/kg/day.

##### Carbohydrates

The infants were started at 7.0–8.0 g/kg/day just after birth. The dose was increased by 1.0–2.0 g/kg/day every day according to the baby’s tolerance to a maximum dose of 15.0–18.0 g/kg/day.

##### Lipids

Intravenous lipid emulsion was started at 0.5 g/kg/day on day two and was increased at a dose of 0.5 g/kg/day according to tolerance to a maximum dose of 3.5 g/kg/day. Beginning in March 2010, Lipo MCT 20% (Dong guk Pharm, Seoul, Korea) was used as the primary parenteral lipid solution. Between April 2011 and January 2013, Intralipid 20% (Fresinius Kabi, Cheshire, UK) was used. Since January 2013, SMOF lipid (Fresenius Kabi, Bad Homburg, Germany) has been used in our NICU. After 2017, Omegaven (Fresinius Kabi, Cheshire, UK) was used as rescue therapy in patients with a diagnosis of PNAC. It was administered twice a week or every other day and was taken with SMOF lipid.

### Statistical analyses

Data are presented as frequencies with percentages for the categorical variables and mean ± standard deviation for continuous variables. Differences in participant characteristics were compared across subgroups with a Chi-square test or Fisher’s exact test for categorical variables and independent test or Mann-Whitney’s U test for continuous variables as appropriate. To check for normal distribution, we used Shapiro-Wilk’s test. Univariate and multivariate analyses, using logistic regression, were performed in order to identify prognostic factors that were independently related to PNAC and death. In addition, the receiver operating characteristic (ROC) curve was performed to assess the sensitivity and specificity for PNAC. All statistical analyses were carried out using SPSS 24.0 and *p* values less than 0.05 was considered statistically significant.

## Results

### Basic demographic and clinical characteristics

**Fig. 1:**
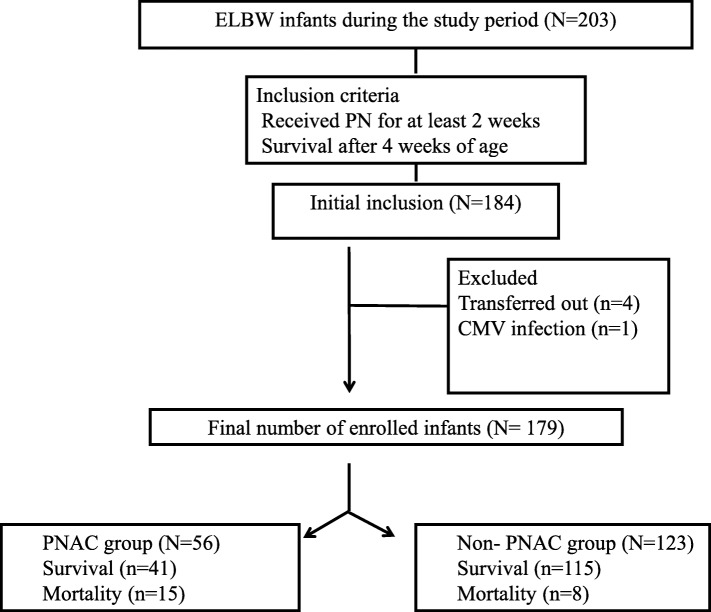
Study flowchart

A total of 203 ELBW infants were admitted during the study period and 184 of these infants met the inclusion criteria. We excluded 5 infants. Four infants were transferred out to other hospital and one infant was diagnosed with an acquired cytomegalovirus infection. Finally, a total of 179 infants were enrolled in this study, and 56 (31.3%) were diagnosed with PNAC at 36.16 ± 17.67 days after birth (Fig. 1). Table 1 shows various clinical factors associated with the development of PNAC. Composition of PN solutions; use of fish-oil based lipid emulsion; and maximum concentrations and cumulative dose of macronutrient including carbohydrate, amino acids, and lipids were not associated with PNAC (data not shown).

**Table 1:** Analysis of risk factors affecting PNAC

Variable	PNAC		
	Yes	No	*P -value*
Gestational age (weeks)			
Mean ± SD	26.07 ± 2.23	26.78 ± 2.32	*.035* ^*2*^
Birth weight (gram)			
Mean ± SD	737.68 ± 151.71	824.72 ± 160.26	*.001* ^*2*^
Hospital days	116.86 ± 56.96	94.34 ± 37.46	*.003* ^*2*^
IUGR			
Yes	20 (35.7)	30 (24.4)	*.117* ^*1*^
No	36 (64.3)	93 (75.6)	
Sex			
Male	27 (48.2)	55 (44.7)	*.663* ^*1*^
Female	29 (51.8)	68 (55.3)	
Apgar score 1 min	4.38 ± 1.61	4.82 ± 1.31	*.085* ^*2*^
Apgar score 5mins	6.70 ± 1.29	6.93 ± 0.98	*.230* ^*2*^
Ventilator duration (days)	55.79 ± 47.23	29.26 ± 25.69	*.000* ^*2*^
PDA			
Surgical ligation	19 (33.9)	22 (17.9)	*.018* ^*1*^
Others	37 (66.1)	101 (82.1)	
ROP			
Laser or bevacizumab inj.	38 (67.9)	59 (48.0)	*.013* ^*1*^
Observation	18 (32.1)	64 (52.0)	
Head ultrasonography			
Normal	35 (62.5)	87 (70.7)	*.273* ^*1*^
Abnormal	21 (37.5)	36 (29.3)	
BPD			
Yes	45 (80.4)	67 (54.5)	*.001* ^*1*^
No	11 (19.6)	56 (45.5)	
NEC			
Yes	27 (48.2)	15 (12.2)	*.000* ^*1*^
No	29 (51.8)	108 (87.8)	
Sepsis			
Yes	40 (71.4)	61 (49.6)	*.006* ^*1*^
No	16 (28.6)	62 (50.4)	
GI surgery			
Yes	19 (33.9)	9 (7.3)	*.000* ^*1*^
No	37 (66.1)	114 (92.7)	
TPN days			
Mean ± SD	74.13 ± 50.62	42.38 ± 31.74	*.000* ^*2*^
Feeding start (days)			
Mean ± SD	4.36 ± 3.25	3.86 ± 2.60	*.234* ^*2*^
Days to achieve full enteral feeding (days)	56.24 ± 22.36	35.06 ± 15.95	*.000* ^*2*^
Type of formula			
Breast milk	20 (37.7)	73 (59.8)	*.007* ^*1*^
Others	33 (62.3)	49 (40.2)	

*Abbreviations: IUGR* intrauterine grow retardation, *PDA* patent ductus arteriosus, *ROP* retinopathy of prematurity, *BPD* bronchopulmonary dysplasia, *NEC* necrotizing enterocolitis, *TPN* total parenteral nutrition, *PNAC* parenteral nutrition–associated cholestasis

^1^*P* values were derived from chi-square test

^2^*P* values were derived from Mann-Whitney’s U test. Shapiro-Wilk’s test was employed for test of normality assumption

### Laboratory test results

**Fig. 2:**
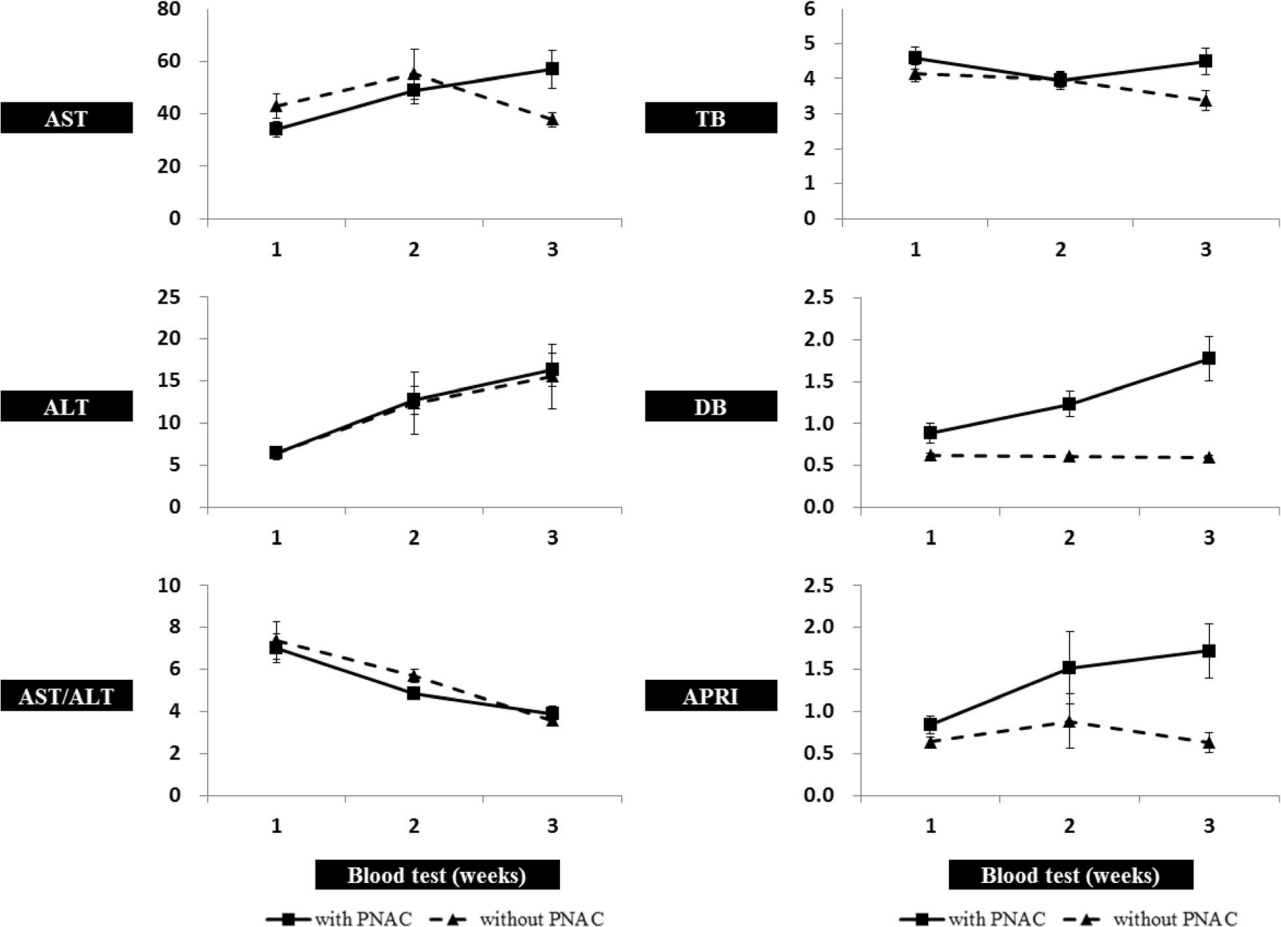
Change in laboratory evaluation at 1, 2, and 3 weeks after parenteral nutrition:  *p < 0.05*,  *p < 0.001*

**Fig. 3:**
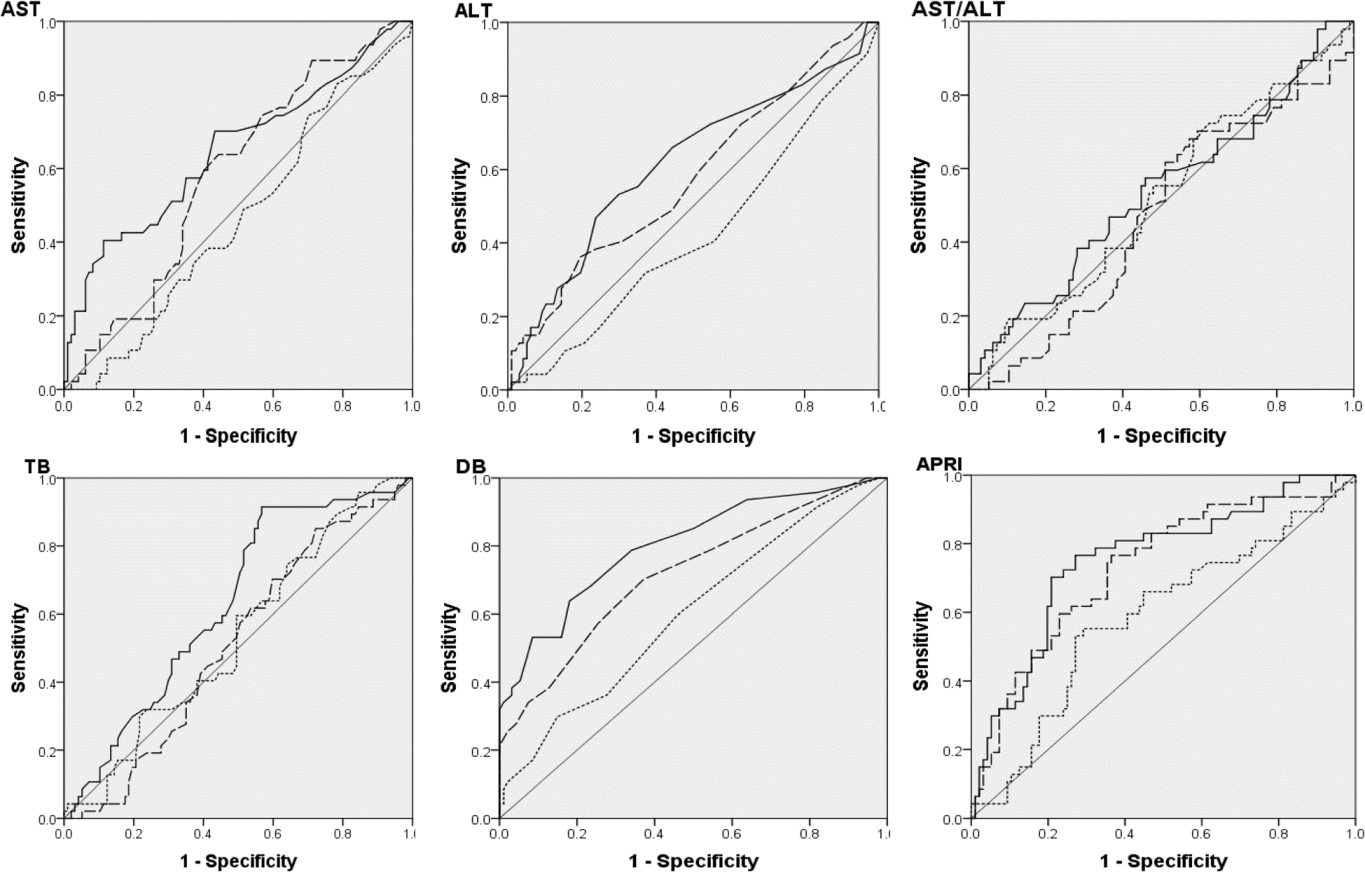
Receiver operating characteristic curves of the laboratory test results to predict PNAC at time of 1 week (dotted line), 2 weeks (dashed line), and 3 weeks after PN (solid line). AUC: area under the curve. *p < 0.05

Figure 2 shows the trends of laboratory test results at 1, 2, and 3 weeks after PN with respect to PNAC. DB and APRI significantly differed between infants with PNAC and without PNAC at each time point and TB and AST also differed between groups at 3 weeks (Table 2). ROC curves for TB, DB, AST, ALT, AST/ALT, and APRI at 1, 2, and 3 weeks of age are presented in Fig. 3.

**Table 2:** Associations with laboratory test results and development of PNAC

Laboratory test results at each time point	PNAC		
	Yes	No	*P -value*
1 week			
AST (mg/dL)	34.14 ± 21.34	43.09 ± 51.40	.821^2^
ALT (mg/dL)	6.41 ± 6.20	6.45 ± 4.56	.414^2^
AST/ALT	7.00 ± 5.27	7.37 ± 9.76	.867^2^
TB (mg/dL)	4.59 ± 2.34	4.16 ± 2.47	.119^2^
DB (mg/dL)	0.89 ± 0.87	0.63 ± 0.24	.015^2^
APRI	0.84 ± 0.81	0.64 ± 0.71	.003^2^
PLT (× 10^3^/μL)	139.98 ± 72.98	191.55 ± 70.45	.000^2^
2 weeks			
AST	48.77 ± 38.47	55.01 ± 99.75	.178^2^
ALT	12.71 ± 12.55	12.32 ± 38.79	.218^2^
AST/ALT	4.84 ± 2.11	5.70 ± 3.28	.394^2^
TB	3.94 ± 1.80	3.98 ± 2.49	.603^2^
DB	1.23 ± 1.14	0.60 ± 0.25	.000^2^
APRI	1.52 ± 3.23	0.89 ± 3.42	.000^2^
PLT	159.75 ± 94.61	263.46 ± 114.66	.000^2^
3 weeks			
AST	57.04 ± 49.33	37.87 ± 28.80	.003^2^
ALT	16.34 ± 13.56	15.52 ± 39.85	.011^2^
AST/ALT	3.91 ± 2.32	3.60 ± 1.99	.569^2^
TB	4.49 ± 2.60	3.38 ± 2.86	.004^2^
DB	1.78 ± 1.83	0.59 ± 0.31	.000^2^
APRI	1.72 ± 2.26	0.63 ± 1.23	.000^2^
PLT	165.10 ± 107.14	285.34 ± 143.80	.000^2^

All values are mean ± standard deviation. APRI (%) = (AST/40)/PLT × 100

Shapiro-Wilk’s test was employed for test of normality assumption

*Abbreviations: TB* total bilirubin, *DB* direct bilirubin, *PLT* platelets, *AST* aspartate aminotransferase, *ALT* alanine transaminase, *APRI* aspartate aminotransferase/platelet ratio index, *PNAC* parenteral nutrition–associated cholestasis

^1^*P* values were derived from chi-square test

^2^*P* values were derived from Mann-Whitney’s U test

### Risk factors associated with PNAC

Figure 3 shows the Receiver operating characteristic (ROC) curves of the DB, APRI to predict PNAC at each time points. Various clinical factors and laboratory test results were associated with the development of PNAC. Finally, the duration of PN and hospitalization, DB and APRI at 2 weeks were determined to be independent risk factors for PNAC in the multivariate logistic regression analysis (Table 3). At 2 weeks after PN, DB > 0.70 and APRI > 0.41 are cut-off points for prediction of PNAC has a 50.0% sensitivity, 91.7% specificity, 75.7% positive predictive value (PPV), and 78.1% negative predictive value (NPV) (Table 4).

**Table 3:** Multiple logistic regression analysis for risk factor of PNAC

Variables	OR	95% CI	*P value*
Hospital day	1.01	(1.00–1.02)	*.023*
Time to full enteral feeding (120 cc/kg/day)	1.06	(1.02–1.09)	*.001*
DB at 2 weeks	23.63	(3.49–160.06)	*.001*
APRI at 2 weeks	3.37	(1.51–7.54)	*.003*

*Abbreviations: APRI* aspartate aminotransferase/platelet ratio index, *DB* direct bilirubin, *PNAC* parenteral nutrition–associated cholestasis

**Table 4:** Predictive values for PNAC of laboratory evaluation at 2 weeks after PN

	Cut-point value	PNAC		Sensitivity%	Specificity%	PPV %	NPV %
		Yes	No				
DB	> 0.7	32	27	57.1%	75.2%	54.2%	77.4%
	≤0.7	24	82				
APRI	> 0.41	43	42	76.8%	62.2%	50.6%	84.1%
	≤0.41	13	69				
DB, APRI	> 0.7 and > 0.41	28	9	50.0%	91.7%	75.7%	78.1%
	≤0.7 or ≤ 0.41	28	100				

*Abbreviations: DB* direct bilirubin, *PNAC* parenteral nutrition–associated cholestasis, *APRI* aspartate aminotransferase/platelet ratio index

## Discussion

PN is essential for survival in preterm infants. Especially, very low birth weight infants have to depend on support of PN for considerable time until establishment of enteral feeding [15]. Among the various clinical features of liver injury results from prolonged PN, PNAC is the most common clinical manifestation in preterm infants [17]. As increase survival of more preterm babies, incidence of PNAC also has increased. Although the precise incidence is still unknown, PNAC has become an emerging topic in neonatal intensive care units (NICUs) [18]. PNAC is an umbrella term that covers a wide spectrum from mild cholestasis or mild elevated liver enzyme to hepatic fibrosis, and cirrhosis. In some cases, the liver undergoes irreversible liver damage, end-stage liver failure, and eventually death [19, 20]. Cyclic or intermittent PN, fish-oil-based lipid emulsion use, and medications including ursodesoxycholic acid are suggested as treatments for PNAC [21, 22]. However, progressed hepatic failure results in mortality. Therefore, it is more important to stop the patient from developing irreversible terminal hepatic failure by preventing liver damage from long term PN. Hence, identification of risk groups for the development of PNAC is essential.

Liver biopsy is currently the gold standard in evaluating liver fibrosis and cirrhosis. However, considering the potential risks in performing liver biopsy, numerous efforts have been made to develop reliable and non-invasive methods for assessment of hepatic fibrosis and cirrhosis. This has led to the introduction of various biological markers, including APRI. Moreover, serial serological markers can be more reliable for reflecting changes of dynamic liver fibrosis. The APRI was initially introduced to monitor and evaluate the progression of fibrosis in adult patients with chronic hepatitis C [23, 24] and it has shown high accuracy in predicting both significant fibrosis and cirrhosis in also hepatitis B [25]. Therefore, it is widely used as a predictive marker to assume the degree of hepatic fibrosis in the adult population with other liver diseases [26, 27]. Moreover, the APRI is an indicator for liver function in end stage liver disease in the younger population [8, 9, 11]. In particular, among infants and children with biliary atresia or a short gut, APRI can predict liver function, liver survival, and prognosis [7, 10]. Because of the differences in characteristics of the enrolled population and variables in the degree of liver fibrosis used as diagnostic criteria, there is a limit to the extent to which specific cut-off value of APRI can be defined. It is clear that levels of APRI are correlated with the progression of hepatic dysfunction and fibrosis in various hepatic diseases in children and adult patients. Only one study evaluated the APRI as a predictor for PNAC in premature infants to date [14]. Underwood et al. assessed 60 infants with gestational age < 34 weeks, birth weight < 2000 g and intestinal perforation due to either NEC or spontaneous intestinal perforation. They reported that APRI was significantly different between 17 infants who later developed PNAC and another 43 infants who did not. They suggested the best APRI cut-point was 0.4775 within 2 weeks after perforation. In the current study, we enrolled ELBW infants on PN regardless of bowel perforation. We checked serial AST, ALT, total bilirubin, direct bilirubin, and APRI at 1, 2, and 3 weeks after birth. Though the differences between infants with PNAC and without PNAC were more prominent with time, considering the statistical significance and values as predictors, we conclude that DB combined with APRI at 2 weeks of age was the most reliable indicator for the development of PNAC in ELBW infants. Because laboratory test results at 1 week had low statistical power and those at 3 weeks could not play a role as predictor; 14 infants were already diagnosed as having PNAC before 3 weeks of age. So, we finally analyzed 2 weeks laboratory test results and finally, the combined vales of DB and APRI has real high specificity; DB > 0.7 and APRI > 0.41 showed a sensitivity of 50.0%, specificity 91.8%, PPV 75.7%, and NPV 78.3%.

Various methods including radiologic work-ups (ultrasound, computed tomography, elastography, magnetic resonance imaging) and serological biomarkers to predict hepatic fibrosis have been proposed. With these method results are encouraging, but it is difficult to perform in preterm infants, especially clinically unstable ELBW infants within the first few weeks. However, platelet counts and basic liver function tests including TB, DB, AST, and ALT are routinely performed in ELBW infants with PN at least once a week. The APRI can be calculated from two routine laboratory tests. Therefore, additional blood sampling or invasive procedures were not needed to evaluate APRI in these neonates, making the test more accessible within the NICU.

This study has some limitations. This study was conducted retrospectively, and therefore various clinical factors including the nutritional protocol, fluconazole prophylaxis and the types of PN solution were not uniformly applied to the study populations. However, these differences allowed us to compare the effects of different clinical factors on the development of PNAC. In addition, we did not perform the live biopsy. Hence, we could not determine a correlation between the biopsy results and APRI values. We also could not determine the exact cut-point value from this single study. However, we do believe that this study suggests the possibility of APRI as an early predictor for PNAC in ELBW infants on PN. Moreover, high specificity, PPV, and NPV of APRI than DB shows the priority of APRI as early predictor.

## Conclusion

This study showed that APRI and DB values at 2 weeks of age had a reliable predictive value for the development of PNAC in ELBW infants. More research on this topic is warranted to help determine the specific values for predicting PNAC to be applied in clinical practice.

## Abbreviations

ALT: Alanine aminotransferase
APRI: AST-to-platelet ratio index
AST: Aspartate aminotransferase
BPD: Bronchopulmonary dysplasia
DB: Direct bilirubin
ELBW: Extremely low birth weight
NEC: Necrotizing enterocolitis
NICU: Neonatal intensive care unit
NPV: Negative predictive value
PN: Parenteral nutrition
PNAC: Parenteral nutrition-associated cholestasis
PPV: Positive predictive value
ROC: Receiver operating characteristic
TB: Total bilirubin
